# Microbiota–Metabolite–Host Crosstalk Mediates the Impact of Dietary Energy Levels on Colonic Homeostasis in High-Altitude Ruminants

**DOI:** 10.3390/ani15192929

**Published:** 2025-10-09

**Authors:** Qinran Yu, Ning Li, Pengjia Bao, Chun Huang, Qingbo Zheng, Tong Wang, Chaofan Ma, Jingying Deng, Fengtao Jiang, Jianlei Jia, Ping Yan

**Affiliations:** 1 Institute of Western Agricultural of CAAS, Changji 831100, China; yuqinran07@163.com (Q.Y.); lining06@caas.cn (N.L.); 13644430207@163.com (C.M.); 2 Institute of Cotton Research, Chinese Academy of Agricultural Sciences, Anyang 455000, China; 3 Lanzhou Institute of Husbandry and Pharmaceutical Sciences, Chinese Academy of Agricultural Sciences, Lanzhou 730050, China; baopengjia@caas.cn (P.B.); johnchun825@163.com (C.H.); zhengqingbo19@163.com (Q.Z.); wgeniust@163.com (T.W.); 17573901120@163.com (J.D.); 4 Kashgar Regional Animal Husbandry Station, Kashgar 844000, China; 13734613889@163.com; 5 School of Life Sciences, Qilu Normal University, Jinan 250200, China

**Keywords:** high-altitude ruminants, dietary energy levels, colonic health, gut microbiota, yaks

## Abstract

**Simple Summary:**

The colon plays a crucial role in maintaining energy metabolism and gut health in ruminants, particularly under extreme high-altitude conditions characterized by hypoxia and cold temperatures. This study involved 60 Pamir yaks that were fed diets with varying energy levels over a period of 170 days to examine the effects of dietary energy on colon health. Our results indicated that higher energy diets enhanced growth performance; however, they also induced colon inflammation and an increase in harmful bacteria. Conversely, the medium-energy diet fostered the proliferation of beneficial microbes, improved immune balance, and regulated key metabolic pathways. These findings suggest that an appropriate level of dietary energy can assist high-altitude ruminants in maintaining colon health through coordinated interactions among microbes, metabolites, and the host.

**Abstract:**

The colon plays a crucial role in energy metabolism and intestinal health of ruminants during various physiological stages. Plateau ruminants have long been subjected to extreme environments characterized by hypoxia, cold, and nutritional scarcity, which makes their dependence on energy metabolism particularly pronounced. However, existing research on the regulatory effects of dietary energy levels on the colonic function of plateau ruminants is still quite limited. This study involved 60 healthy male Pamir yaks with consistent body conditions, which were randomly divided into three groups: a low-energy diet group (YG, Neg 1.53 MJ/kg), a medium-energy diet group (QG, Neg 2.12 MJ/kg), and a high-energy diet group (RG, Neg 2.69 MJ/kg). Each yak was provided with 5 kg of mixed feed daily over a 170-day feeding trial. The results indicated that a high-energy diet enhanced growth performance in yaks (*p* < 0.05). However, it also induced local colonic inflammation, decreased levels of immune factors (IgA, IgG, and IL-10), and increased the abundance of potentially pathogenic bacteria, such as *Klebsiella* and *Campylobacter* (*p* < 0.05). Conversely, a medium-energy diet fostered the proliferation of beneficial bacteria such as *Bradymonadales*, *Parabacteroides*, and *Mogibacterium* (*p* < 0.05), and preserved immune homeostasis. Additionally, multi-omics analysis revealed that the QG group was significantly enriched in key metabolic pathways, including pyruvate metabolism and glycine, serine, and threonine metabolism and panto-thenate and CoA biosynthesis pathways, among others (*p* < 0.05), demonstrating a synergistic regulatory effect among the microbiome, metabolism, and host. In summary, a moderate-energy diet can promote the proliferation of beneficial bacteria in the extreme environment of the plateau. By regulating pathways such as Amino acid, Nucleotide, and Lipid metabolism, it coordinates the expression of key host genes and metabolite levels, effectively balancing immune signals and energy metabolism. This interaction establishes a beneficial microbial-metabolism-host pattern that supports colon health.

## 1. Introduction

In high-altitude regions (>3000 m), ruminants encounter severe environmental challenges, including rarefied air, high elevations, and substantial diurnal temperature fluctuations. As altitude rises, atmospheric pressure diminishes progressively, resulting in a continuous decrease in the partial pressure of oxygen (PO_2_) and consequently inducing a hypoxic state. Simultaneously, environmental temperatures decline with increasing altitude, with an approximate temperature drop of 6.5 °C for every 1000 m of elevation gain [1]. In such environments, the amount of oxygen that ruminants obtain with each breath is only approximately 60% of that available at sea level. This inadequate oxygen supply not only limited the efficiency of aerobic metabolism but also presented significant challenges to sustaining normal energy metabolism and physiological functions within the body [2,3]. To adapt to this environment, typical plateau ruminants such as Tibetan sheep, yaks, and Tibetan antelopes have developed unique physiological and genetic adaptations over long-term evolution, which enable them to survive and reproduce in the cold and hypoxic conditions of high altitudes. However, relying solely on their own regulatory mechanisms remains insufficient to fully withstand the constraints imposed by this extreme environment. Moreover, plateau regions frequently experience harsh weather conditions, characterized by a limited supply of natural forage and a notable decline in nutritional quality. Sole reliance on grazing has proven inadequate to satisfy the energy requirements necessary for animal growth and production. Consequently, in recent years, confined feeding and supplementary feeding models have gained popularity, emerging as essential strategies to ensure the health and production of ruminants at high altitudes.

The regulation of dietary energy levels not only affects host metabolism but may also contribute to the host’s environmental adaptation by reshaping the gut microbiota and their metabolites [4,5,6,7]. The gut microbiota, a crucial component of energy metabolism and host homeostasis in ruminants, played a significant role in regulating metabolic processes, protecting the host from pathogenic microorganisms, and modulating the immune system [8,9,10,11]. Gut microbiota can generate a variety of metabolites through the fermentation of dietary components. These metabolites not only regulated the host’s metabolic pathways but also influenced its physiological and pathological states [12]. Among these metabolites, short-chain fatty acids (SCFAs) were representative products of microbial metabolism. SCFAs provide the host with additional energy sources and play a crucial role in regulating energy metabolism, immune responses, and gut barrier function through various signaling pathways [13]. In ruminants, the hindgut microbiota played an irreplaceable role in nutrient absorption, metabolism, gastrointestinal development, and immune function [14,15]. Especially during the juvenile stage, the colon serves as a primary ecological niche for microbial colonization and fermentation, with its metabolic activities being crucial for the host’s energy metabolism, immune maturation, and physiological development [16]. Furthermore, early-life feeding strategies significantly influence both the composition of the colonic microbiota and its transcriptomic characteristics [17,18]. Despite the growing interest in the role of gut microbiota in energy metabolism, existing research has predominantly concentrated on the early developmental stages of calves. Systematic studies investigating the interaction mechanisms between colonic microbiota and their host in adult plateau ruminants are still quite limited, especially concerning the mechanisms of energy metabolism, which necessitate further in-depth exploration.

Based on the aforementioned background, we hypothesize that different dietary energy levels alter the composition of the colonic microbiota and the profile of its metabolites. These changes may regulate the expression of host genes related to colonic function and thereby influence the maintenance of colonic tissue homeostasis. Furthermore, this gut–host interaction may ultimately modulate overall growth performance and the ability of the animals to adapt to environmental stress. Yaks (*Bos grunniens*), as the primary livestock species on the Qinghai–Tibet Plateau, thrive in harsh environments characterized by extreme cold (with an average annual environmental temperature close to 0 °C), high altitude (over 3000 m), and hypoxia (with oxygen levels below 70% of those at sea level) [19,20]. This makes it an ideal model for studying intestinal health and the regulatory mechanisms of energy metabolism in plateau ruminants. This study focuses on the Pamir yak as the research subject, establishing three dietary energy levels: low energy (YG), medium energy (QG), and high energy (RG). Through 16S rRNA sequencing, serum and colon metabolomics, colon transcriptomics, and SCFA analysis, combined with a multi-omics comprehensive analysis of colon immune and antioxidant indicators. This study systematically evaluates the regulatory effects of different energy levels on colon microbiota, metabolism, and host transcriptional responses. The research aims to elucidate how appropriate energy levels promote the maintenance of colon homeostasis and adaptation to the plateau environment in yaks through the microbial–metabolite–host interaction mechanism, thereby providing a theoretical basis for precise nutritional management and health of plateau ruminants.

## 2. Materials and Methods

### 2.1. Animal Experiment

This study was conducted at a natural pasture in Taxkorgan Tajik Autonomous County, Kashgar Prefecture, Xinjiang, China (geographic coordinates: 37.78° N, 75.22° E, approximately 3800 m above sea level). Sixty healthy, uniformly sized male yaks (initial body weight 260 ± 13.29 kg and age 3.0 years) were selected and randomly divided into three groups of 20 yaks each. The experiment comprised three diets with varying energy levels: Group 1 (YG) was administered a low-energy diet (NEg energy 1.53 MJ/kg, 5 kg of mixed diet per yak per day); Group 2 (QG) received a medium-energy diet (NEg energy 2.12 MJ/kg, 5 kg of mixed diet per yak per day); and Group 3 (RG) was provided a high-energy diet (NEg energy 2.69 MJ/kg, 5 kg of mixed diet per yak per day). The entire experimental period spanned 170 days, from 1 November 2023 to 18 April 2024. The initial 20 days served as an acclimatization phase, during which the proportion of concentrate feed was gradually increased. The subsequent 150 days constituted the official data collection phase. Yaks were fed twice daily at 10:00 AM and 6:00 PM, with unrestricted access to their designated rations and clean drinking water throughout the feeding schedule. Body weight measurements were performed at birth (day 0) and at 1, 2, 3, and 4 months of age. This feeding regimen was informed by the feed intake of yaks grazing on the Pamir Plateau and adheres to the “Chinese Beef Cattle Feeding Standard” (NY/T 815-2004) [21]. The concentrate feed utilized in the experiment was custom-produced by Tianjian Agricultural Technology Co., Ltd. (Shandong, China), while the roughage consisted of wheat straw, mixed in equal proportions. The chemical composition and nutritional quality of the diets were analyzed by the Qilu Normal University Laboratory, following the “Feed Analysis and Quality Testing Technology” standard. Detailed results can be found in Supplementary Materials Table S1.

### 2.2. Sample Collection

Following the experiment, all yaks were subjected to a 24 h fasting period and deprived of water for 8 h. Subsequently, three yaks were randomly selected from each group, anesthetized using a bullet gun, and sacrificed via carotid exsanguination. Blood samples were collected during exsanguination at slaughter and collected into 5 mL vacuum-free blood collection tubes (Jiangsu Yuli Medical Instruments Co., Ltd., Jiangyan, China). To facilitate complete clotting and efficient serum separation, the blood samples were incubated in a 37 °C water bath for 15 min and then centrifuged at 3500× *g* for 15 min at 4 °C to collect serum for subsequent metabolomics analysis. Following slaughter, colonic contents were collected and immediately snap-frozen in liquid nitrogen. Samples were subsequently used for 16S rRNA sequencing, metabolomics analysis, short-chain fatty acid determination, and assessment of colonic immune indicators [22,23]. Colonic tissue was obtained from the mid-colon, approximately 6 cm per yak, and was rinsed three times with ice-cold sterile 0.01 M PBS buffer (pH 7.2–7.4, Beijing Solar Biotechnology Co., Ltd., Beijing, China) within 5 min post-slaughter. A portion of the tissue was snap-frozen in liquid nitrogen and stored at −80 °C for transcriptomic analysis, while the remaining portion was fixed in 4% paraformaldehyde, subsequently dehydrated, trimmed, embedded, sectioned, and stained with hematoxylin and eosin (H&E), followed by sealing to prepare sections for histopathological examination.

### 2.3. Histological Analysis

Colonic H&E-stained sections were used for the grading of colonic inflammation [16,24]. In brief, 0, no signs of inflammation; 1, very low level; 2, low level of leukocytic infiltration; 3, high level of leukocytic infiltration, high vascular density, thickening of the colon wall; 4, transmural infiltrations, loss of goblet cells, high vascular density, thickening of the colon wall. About 3 images per sample were taken with Nikon ECLIPSE Ni-U (Nikon Co., Ltd., Tokyo, Japan) at ×100 magnification, and the average scores of each sample were calculated.

### 2.4. Colon Immune Index Determination

The indicators selected were IgA, IgG, IL-6, IL-1β, TNF-α, IL-10, and IL-8; enzyme-linked immunosorbent assay (ELISA) kits (Jiangsu ELISA Industrial Co., Ltd., (Wuxi, China)) were used for determination, and the determination methods were carried out according to the instructions.

### 2.5. Total RNA Extraction and Sequencing

Total RNA was extracted from colonic contents samples using TRIzol reagent, following the manufacturer’s instructions. RNA purity was assessed with a NanoDrop ND-2000 spectrophotometer (Thermo Scientific, Waltham, MA, USA). The quality of the extracted RNA was evaluated using an Agilent 2100 Bioanalyzer (Agilent Technologies Inc., Santa Clara, CA, USA), ensuring that all samples had a RIN > 8. Transcriptome libraries were constructed using the VAHTS Universal V10 RNA-seq Library Prep Kit (Premixed Version; Vazyme Biotech Co., Ltd., Nanjing, China), in accordance with the manufacturer’s guidelines. The library was sequenced using the Illumina NovaSeq 6000 platform, producing 150 bp paired-end reads. The raw reads in FASTQ format were processed with the fastp (v0.23.2) [25] to eliminate low-quality reads, yielding clean reads for subsequent data analysis. Reference genome alignment was performed using HISAT2 (v2.2.1) [26], and gene expression levels were calculated as FPKM [27]. Read counts for each gene were obtained through HTSeq-count (v0.13.5) [28]. Principal Component Analysis (PCA) and gene count plotting were carried out using R (v3.2.0) to assess the biological replicates of the samples. Differential expression analysis was conducted using DESeq2 (v1.36.0) [29], where genes with a q-value < 0.05 or a fold change <0.5 were classified as differentially expressed genes (DEGs). KEGG [30] pathway enrichment analysis of the DEGs was performed based on the hypergeometric distribution algorithm to identify significantly enriched functional categories. R (v3.2.0) was also utilized to create Sankey interaction diagrams for the significantly enriched functional categories.

### 2.6. Microbial DNA Extraction

Bacterial genomic DNA was extracted using the DNeasy PowerSoil DNA extraction kit (Qiagen, Hilden, Germany) in accordance with the manufacturer’s instructions and subsequently stored at −20 °C for future use. The concentration and purity of the DNA were assessed using a NanoDrop 2000 spectrophotometer (Thermo Fisher Scientific, USA) and 1% agarose gel electrophoresis, respectively.

### 2.7. PCR Amplification and 16S rRNA Gene Sequencing

A total of nine colonic contents samples were subjected to 16S rRNA gene sequencing. Bacterial genomic DNA was extracted with the DNeasy PowerSoil kit (Qiagen, Hilden, Germany) according to the manufacturer’s instructions and the extracts were stored at −20 °C until further use. DNA concentration and purity were assessed with a NanoDrop 2000 spectrophotometer (Thermo Fisher Scientific, Waltham, MA, USA) and by 1% agarose gel electrophoresis. The V3-V4 hypervariable region of the bacterial 16S rRNA gene was amplified using PCR with the universal primer pair 343F (5′-TACGRAGGCAGCAG-3′) and 798R (5′-AGGGTATCTAATCCT-3′) [31]. Subsequently, 250 bp paired-end sequencing was conducted on the Illumina NovaSeq 6000 platform. Raw sequencing data were obtained in FASTQ format. Following sequencing, primer sequences were initially removed from the raw data using Cutadapt (v3.4). Subsequently, quality control was performed on the trimmed paired-end sequences, which included filtering low-quality sequences, denoising, pairwise merging, and chimera removal. This quality control process was conducted using the QIIME 2 platform, specifically the DADA2 plug-in, along with its default parameters [28,32]. After quality control, characteristic sequences (ASVs) and their corresponding abundance tables were generated. Representative sequences for each ASV were extracted using QIIME 2, and the q2-feature-classifier plug-in was employed for classification and annotation in the Silva database (version 138).

Alpha and beta diversity of the samples were assessed using the QIIME 2 (v2022.8) package. Alpha diversity was measured using the Shannon index, Chao1 index, and ACE index to measure species richness and diversity. Intergroup differences in alpha diversity were analyzed using the Wilcoxon rank-sum test. Beta diversity was assessed by principal coordinates analysis (PCoA) based on Bray–Curtis distances to visualize inter-sample variation, and the significance of community-structure differences among groups was further evaluated using PERMANOVA (Adonis test). To identify differential bacterial genera among the three dietary energy groups, the relative abundance of microbial taxa at the genus level was compared using the Kruskal–Wallis test, with Benjamini–Hochberg false discovery rate (FDR) correction; genera with an adjusted q-value < 0.05 were considered significant. Linear discriminant analysis effect size (LEfSe) analysis (LDA > 2) was used to screen bacterial groups with significant differences in abundance between groups from the phylum to genus level (*p* < 0.05). Functional predictions of 16S sequences were conducted using PICRUSt2 (version 2.2.0), and intergroup differences in predicted pathways were analyzed with STAMP, applying Benjamini–Hochberg FDR correction (significance set at FDR < 0.05).

### 2.8. Untargeted Metabolomics and Targeted Short-Chain Fatty Acid Profiling

We performed both untargeted metabolomics and targeted SCFA profiling of the same samples used for 16S rRNA sequencing. Untargeted metabolomics were performed using a high-resolution LC-MS/MS platform (Thermo Fisher Scientific). Raw data were processed, and metabolite analysis was conducted using Progenic QI V2.3 software (Nonlinear Dynamics, Newcastle, UK). Metabolite identification relied on annotations from multiple databases, including the Human Metabolite Database (HMDB), LipidMaps (V2.3), Metlin database, and a custom-built database. Key parameters included a precursor ion tolerance of 5 ppm, a product ion tolerance of 10 ppm, and a product ion threshold of 5%. For metabolomics, partial least squares discriminant analysis (PLS-DA) was performed to visualize overall clustering. Metabolomics data were further analyzed using orthogonal PLS-DA (OPLS-DA) to calculate variable importance in projection (VIP) scores. Metabolites with |FoldChange| > 1, VIP > 1, and FDR-adjusted *p* value < 0.05 were considered significantly different. More detailed information was provided in the Supplementary Materials File S1. For SCFA profiling, we initially prepared a standard solution. Subsequently, 20 mg of the fecal sample was weighed into a 2 mL grinding tube, and 800 μL of 0.5% phosphoric acid (containing the internal standard 2-ethylbutyric acid at 10 μg/mL) was added. The sample was cryo-ground for 3 min at 50 Hz, followed by sonication for 10 min and centrifugation at 13,000× *g* for 15 min at 4 °C. A volume of 200 μL of the supernatant was carefully removed and transferred to a 1.5 mL centrifuge tube, followed by extraction with 200 μL of n-butanol. The sample was vortexed for 10 s, sonicated for an additional 10 min, and then centrifuged at 13,000× *g* for 5 min at 4 °C. The supernatant was subsequently transferred to a vial and analyzed using a GC-MS system. GC-MS analysis was performed with a GC/MSD gas spectrometer (Agilent Technologies Inc., Santa Clara, CA, USA, 8890-7000D). The Masshunter quantitation software (Agilent, USA, version 10.0.707.0) was utilized with default parameters to automatically identify and integrate the fragment ions of the target short-chain fatty acids, with manual verification conducted as necessary. The concentration of each sample was calculated using a standard curve to determine the actual content of short-chain fatty acids in the sample. Additional details are provided in the Supplementary Materials File S1.

### 2.9. Validation of Differentially Expressed Genes Using qPCR Analysis

Colonic total RNA of all slaughtered and sampled yaks was reverse transcribed using Prime Script™ RT Master Mix (Takara, Inc., Beijing, China). qPCR was conducted on iCycler IQTM5 (Bio-Rad, Inc., Hercules, CA, USA) using SYBR green dye (TB Green^®^ Premix Ex Taq™ II Tli RNaseH Plus, Takara, China) following the standard program [33]. The data of the gene expression were normalized to the housekeeping gene (β-actin) using the 2^−ΔΔCT^ method [34].

### 2.10. Statistical Analysis

For phenotypic traits, total body weight gain was expressed as mean ± standard deviation (SD). Data normality was assessed by the Shapiro–Wilk test. Between-group differences in total weight gain were analyzed by one-way analysis of variance (ANOVA) followed by Tukey’s post hoc test. Differences were considered statistically significant at *p* < 0.05. Similarly, colonic histopathological scores, colonic immune parameters, SCFAs analysis and qRT-PCR result verification were analyzed using the same statistical approach. Correlation analysis between Differential bacterial communities and other parameters was performed using Spearman’s rank correlation, and correlations with FDR < 0.1 were considered significant.

## 3. Results

### 3.1. Growth Performance, Colonic Pathology, and Immune Responses

**Figure 1:**
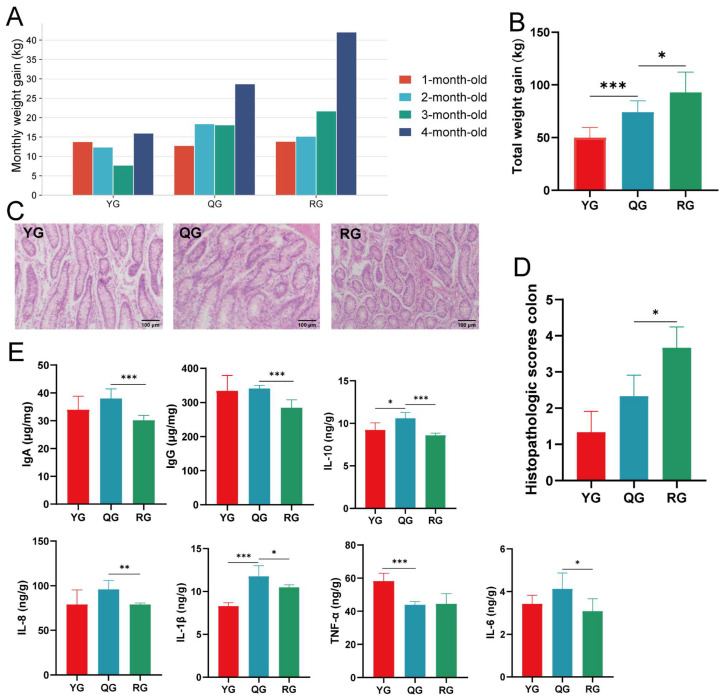
(**A**) Monthly body weight gain of yaks during the fattening period across three dietary treatments. (**B**) Total body weight gain during the fattening period. (**C**) Representative H&E-stained colonic sections from the three treatment groups. (**D**) Histopathological scores of colonic tissues (*n* = 3). (**E**) Colonic immune indices. Symbols indicate statistical significance (* *p* < 0.05, ** *p* < 0.01, *** *p* < 0.001).

Figure 1A shows the monthly weight gain of YG, QG, and RG groups during the four-month fattening period. No significant difference was observed among the three groups in the first month. Thereafter, the RG group exhibited a continuous increase, reaching the highest gain in the fourth month and being significantly higher than the QG and YG groups (*p* < 0.05), with the QG group remaining intermediate and the YG group consistently lowest. Over the entire fattening period (Figure 1B), the RG group achieved the highest total weight gain, followed by QG and YG (*p* < 0.05). Colonic pathology scoring (Figure 1C,D) revealed that the YG group had regular glandular arrangement, the presence of goblet cells, and mild inflammatory cell infiltration. The QG group had a generally regular glandular pattern overall, but with an increased number of inflammatory cells, moderate inflammation in the lamina propria, and mild irregular morphology in some glands. In the RG group, the glandular pattern was densely packed or distorted in some areas. The intergroup differences in scores were statistically significant between the RG and QG groups (*p* < 0.05). Subsequently, we found that IgA, IgG, and IL-10 levels were highest in the QG group and significantly decreased in the RG group (*p* < 0.001). IL-8, IL-1β, and IL-6 also showed similar trends, all of which were higher in the QG group than in the RG group (*p* < 0.05). Notably, the TNF-α content was highest in the YG group, significantly higher than in the QG and RG groups (*p* < 0.05) (Figure 1E).

### 3.2. 16S rRNA Analysis Revealed That Energy Levels Alter the Colonic Microbiome

**Figure 2:**
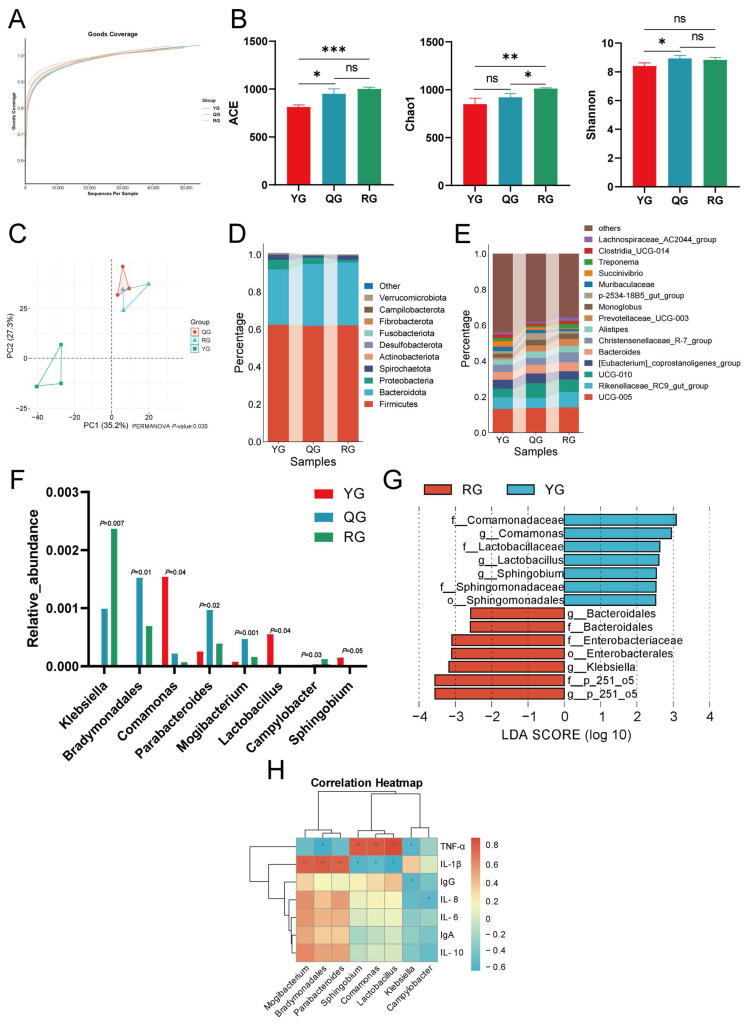
(**A**) Good’s coverage estimates across all samples. The coverage values exceeded 99% for each sample, indicating sufficient sequencing depth to capture the majority of microbial diversity present. (**B**) Alpha-Diversity indices: ACE index, Chao1 index and Shannon index. Data = mean ± SD; * *p* < 0.05, ** *p* < 0.01, *** *p* < 0.001 (one-way ANOVA with Tukey’s post hoc test). (**C**) PCoA of YG, QG, and RG groups based on Bray-Curtis dissimilarity (PC1/PC2 shown). (**D**) Phylum-level community composition (top 10 taxa). (**E**) Genus-level community composition (top 15 genera). (**F**) The abundance of differential microbiota screened from these three treatment groups (one-way ANOVA with Tukey’s post hoc test). (**G**) Differentially abundant genera across YG, QG, and RG groups (LDA score > 4). (**H**) Correlation heatmap between differential bacterial flora and colonic immune indicators. Rows and columns represent colonic immune indicators and differential bacterial flora, respectively, and cell color indicates the Spearman correlation coefficient. Significance levels: * *p* < 0.05, ** *p* < 0.01, *** *p* < 0.001, ns = not significant.

To investigate the effects of diets with varying energy levels on the colonic microbiome of yaks, this study performed high-throughput 16S rRNA sequencing of colonic samples. Good’s coverage for all samples exceeded 0.99 (Figure 2A), indicating that the sequencing depth was sufficient to capture most microbial taxa and ensure the reliability of subsequent alpha diversity analyses. Alpha diversity analysis revealed significant differences in microbial richness and diversity among the various groups (Figure 2B). The ACE index indicated that the QG group exhibited a significantly higher score compared to the YG group (*p* < 0.05). However, no significant difference was observed between the QG and RG groups. In contrast, the difference between the RG and YG groups was found to be extremely significant (*p* < 0.001). The Chao1 index revealed no significant difference between the YG and QG groups; however, the RG group demonstrated a significantly higher score than the QG group (*p* < 0.05). Analysis of the Shannon index showed a significant difference between the YG and QG groups (*p* < 0.05), while no significant differences were detected between the QG and RG groups or between the RG and YG groups. Furthermore, beta-diversity PCoA analysis indicated that the YG, QG, and RG groups displayed distinct separations in community structure, with minor overlap observed between the QG and RG groups (Figure 2C). This separation was statistically supported by PERMANOVA, showing a significant difference among groups (*p* < 0.035), indicating that dietary energy levels influenced the colonic microbial community structure. At the taxonomic level, the relative abundance of the colonic microbiota of yaks in the different energy groups showed significant differences. Phylum-level analysis showed that Firmicutes and Bacteroidetes were the dominant phyla in the three groups, with Proteobacteria and Spirochaetes also detected in small amounts (Figure 2D). At the genus level, the dominant genera in each group were mainly *UCG-005*, *Rikenellaceae_RC9_gut_group*, *UCG-010*, *[Eubacterium]_coprostanoligenes_group*, *Bacteroides*, *Christensenellaceae_R-7_group*, *Alistipes* and *Prevotellaceae_UCG-003* (Figure 2E). The microbial genus samples from the three treatment groups were analyzed using the Kruskal–Wallis test. The results showed that 8 differential microorganisms were screened out among the three treatment groups, including *Klebsiella*, *Bradymonadales*, *Comamonas*, *Parabacteroides*, *Mogibacterium*, *Lactobacillus*, *Campylobacter* and *Sphingobium*. Figure 2F indicated that the levels of *Klebsiella* and *Campylobacter* were significantly elevated in the RG group compared to the YG and QG groups (*p* < 0.05). In contrast, the QG group showed significantly higher levels of *Bradymonadales*, *Parabacteroides*, and *Mogibacterium* when compared to the YG and RG groups (*p* < 0.05). Additionally, the YG group exhibited significantly greater levels of *Comamonas*, *Lactobacillus*, and *Sphinobium* relative to the QG and RG groups (*p* < 0.05). These differences indicated that the colonic microbiota composition was reshaped with increasing energy levels. LEfSe analysis showed that the enriched bacterial groups in the YG group were mainly *f_Comamonadaceae*, *g_Comamonas*, *f_Lactobacillaceae*, *g_Lactobacillus*, *g_Sphingobium*, *f_Sphingomonadaceae*, and *o_Sphingomonadales*; the enriched bacterial groups in the RG group were *g_p_251_o5*, *f_p_251_o5*, *g_Klebsiella*, *o_Enterobacterales*, *f_Enterobacteriaceae*, *f_Bacteroidales*, and *g_Bacteroidales*. Notably, no unique bacterial groups were found to be significantly enriched in the QG group (Figure 2G). A correlation analysis was performed to evaluate the relationship between the varying bacterial communities and colonic immune markers (Figure 2H). Our results indicated that there were significant positive correlations of *Mogibacterium*, *Bradymonadales*, and *Parabacteroides* with IL-1β (*p* < 0.01). Additionally, *Sphingobium*, *Comamonas*, and *Lactobacillus* exhibited significant positive correlations with TNF-α (*p* < 0.01) and significant negative correlations with IL-1β (*p* < 0.05). Moreover, *Klebsiella* displayed significant negative correlations with both TNF-α and IgG (*p* < 0.05).

**Figure 3:**
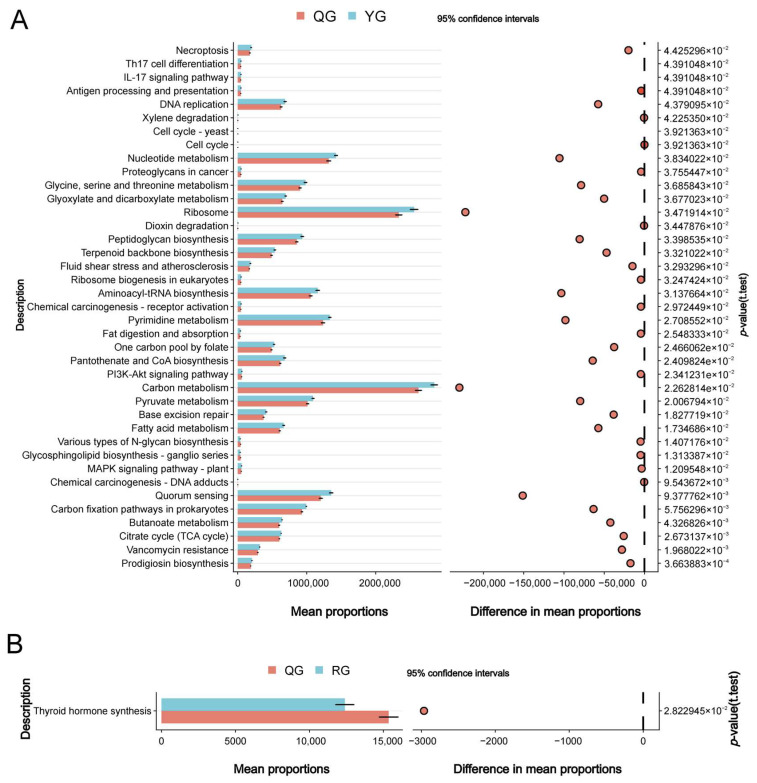
KEGG enrichment analysis of metabolic pathways. (**A**) Show the differential analysis of metabolic pathways between the YG and QG groups. (**B**) Show the differential analysis of metabolic pathways between the QG and YG groups.

Based on 16S rRNA sequencing data, PICRUSt2 was used to predict the function of the gut microbial community. Differential analysis at KEGG level 3 showed that metabolic pathways such as carbon metabolism, ribosome metabolism, pyrimidine metabolism, and nucleotide metabolism were significantly enriched in the YG and QG groups (*p* < 0.05) (Figure 3A). In contrast, only thyroid Hormone synthesis was significantly enriched in the QG and RG groups (*p* < 0.05) (Figure 3B).

### 3.3. Responses of Serum Metabolic Profiles to Energy Levels

**Figure 4:**
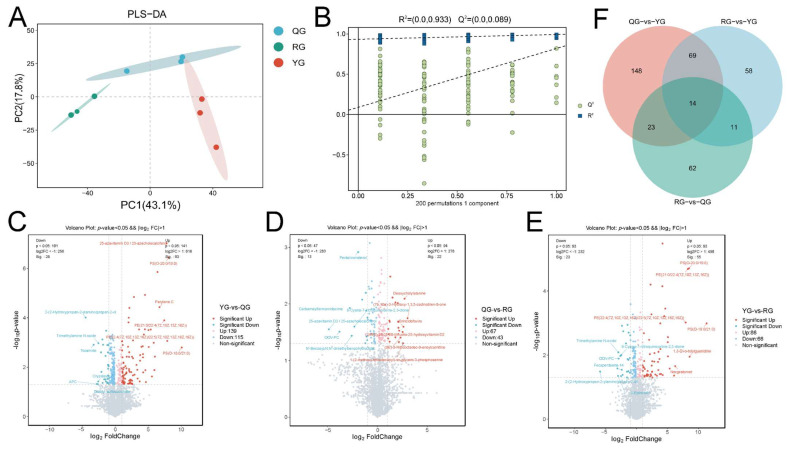
(**A**) OPLS-DA score plots of serum metabolite data between the three comparison groups. (**B**) Corresponding orthogonal partial least squares discriminant analysis model evaluation parameters (R^2^ and Q^2^) are used to assess the model’s fitting and predictive ability. The dashed lines represent the regression lines of the R^2^ and Q^2^ values obtained from 200 permutation tests. (**C**) Venn diagram of shared metabolites across treatment groups. (**D**,**E**) Volcano plots of differential metabolites: (**D**) YG vs. QG; (**E**) QG vs. RG; (**F**) YG vs. RG. Red dots represent significantly upregulated metabolites, blue dots represent significantly downregulated metabolites, and gray dots represent metabolites with no significant difference, with screening criteria of *p* < 0.05 and VIP > 1.0.

To evaluate the effects of different energy levels on whole-body metabolism in yaks, we first analyzed the changes in serum metabolites. The PLS-DA results indicated a distinct separation trend in the serum metabolic profiles among the different energy groups (Figure 4A). In the PLS-DA validation model, the intercept of the Q^2^ regression line was less than zero, suggesting that the model was reliable and not overfit (Figure 4B). Furthermore, by applying the thresholds of FC  >  1, VIP  >  1, and *p*  <  0.05, a total of 254 differential metabolites were identified between the YG and QG groups, including 93 significantly upregulated and 28 significantly downregulated metabolites. These metabolites were primarily classified into lipids and lipid-like molecules, organic acids and derivatives, organoheterocyclic compounds, benzenoids, organic oxygen compounds, phenylpropanoids and polyketides (Figure 4C). A total of 110 distinct metabolites were found when comparing the QG group with the RG group, which included 22 metabolites that showed significant upregulation and 13 metabolites that exhibited significant downregulation. The primary categories of these metabolites comprised Lipids and lipid-related molecules, Organic acids and their derivatives, Organoheterocyclic compounds, and Benzenoids (Figure 4D). Additionally, a total of 152 distinct metabolites were discovered in the comparison between the YG group and the RG group, with 55 metabolites significantly upregulated and 23 metabolites significantly downregulated. It mainly includes Lipids and lipid-like molecules, Organic acids and derivatives, Organoheterocyclic compounds, Benzenoids, and Organic oxygen compounds (Figure 4E). Furthermore, we identified 117 metabolites that were shared among the treatment groups (Figure 4F).

**Figure 5:**
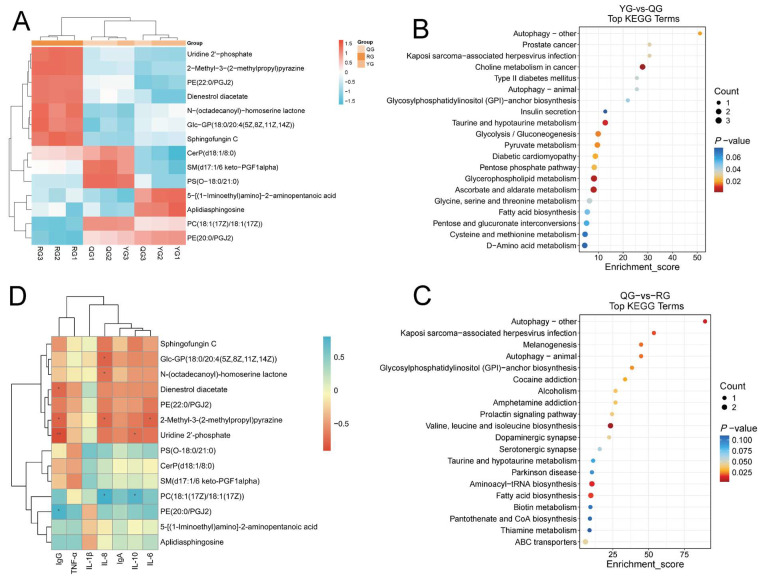
(**A**,**B**) KEGG pathway enrichment bubble charts (top 20 pathways): (**A**) YG vs. QG; (**B**) QG vs. RG; *Y*-axis: names of enriched metabolic pathways; *X*-axis: enrichment factor; bubble size: number of metabolites involved; color gradient: −log_10_ (*p*-value). (**C**) Clustered heatmap of 14 key metabolites; color gradient indicates relative abundance levels across groups. (**D**) Correlation heatmap between serum differential metabolites and colonic immune indicators. Rows and columns represent serum differential metabolites and immune indicators, respectively, and cell color indicates the Spearman correlation coefficient. Significance levels: * *p* < 0.05, ** *p* < 0.01.

Based on this, we constructed a clustered heatmap of the 14 differential metabolites shared by the three comparison groups to visually illustrate the clustering patterns of metabolites under different treatment conditions (Figure 5A). KEGG pathway enrichment analysis revealed that the YG group and QG group were significantly enriched in 15 pathways, including choline metabolism in cancer, glycerophospholipid metabolism, and ascorbate and aldarate metabolism (Figure 5B). The QG group and RG group were significantly enriched in 14 pathways, including valine, leucine and isoleucine biosynthesis, aminoacyl-tRNA biosynthesis, and fatty acid biosynthesis (Figure 5C). Subsequently, we conducted a correlation analysis between the serum differential metabolites and immune indicators. As shown in Figure 4D, Dienestrol diacetate, 2-Methyl-3-(2-methylpropyl) pyrazine, and Uridine 2′-phosphate exhibited a significant positive correlation with IgG (*p* < 0.05). Conversely, PE (20:0/PGJ2) showed a significant negative correlation with IgG (*p* < 0.05). Glc-GP (18:0/20:4 (5Z, 8Z, 11Z, 14Z)), N-(octadecanoyl)-homoserine lactone, and 2-Methyl-3-(2-methylpropyl) pyrazine showed significant positive correlations with IL-8 (*p* < 0.05). 2-Methyl-3-(2-methylpropyl) pyrazine also showed a significant positive correlation with IL-6 (*p* < 0.05). Uridine 2′-phosphate similarly exhibited a significant positive correlation with IL-10 (*p* < 0.05). PC (18:1 (17Z)/18:1 (17Z)) showed a significant negative correlation with IL-8 and IL-10 (*p* < 0.05).

### 3.4. Analysis of Local Metabolic Differences and Short-Chain Fatty Acids in the Colon Under Different Energy Treatments

**Figure 6:**
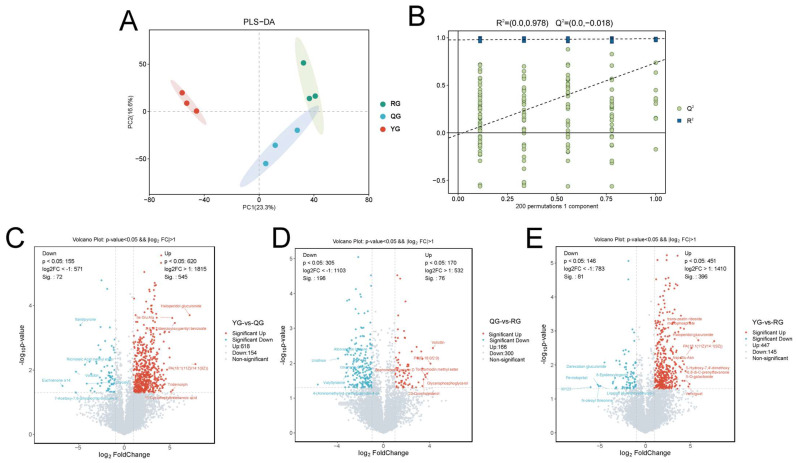
(**A**) OPLS-DA score plots of colonic metabolite data between the three comparison groups. (**B**) Corresponding orthogonal partial least squares discriminant analysis model evaluation parameters (R^2^ and Q^2^) are used to assess the model’s fitting and predictive ability. The dashed lines represent the regression lines of the R^2^ and Q^2^ values obtained from 200 permutation tests. (**C**–**E**) Volcano plots of differential metabolites: (**C**) YG vs. QG; (**D**) QG vs. RG; (**E**) YG vs. RG; Red dots represent significantly upregulated metabolites, blue dots represent significantly downregulated metabolites, and gray dots represent metabolites with no significant difference, with screening criteria of *p* < 0.05 and VIP > 1.0.

Subsequently, to further investigate the effects of different energy levels on the colonic metabolism of yaks, we conducted metabolomic analysis and short-chain fatty acid determination on the colonic contents. The PLS-DA results indicated a certain separation trend in the colonic metabolic profiles among the three treatment groups (Figure 6A). In the PLS-DA validation model, the intercept of the Q2 regression line was less than 0, indicating that the model was reliable and not overfit (Figure 6B). Differential analysis revealed that a total of 772 differential metabolites were identified between the YG group and the QG group, with 545 significantly upregulated and 72 significantly downregulated; 466 differential metabolites were identified between the QG group and the RG group, with 76 significantly upregulated and 198 significantly downregulated; and 592 differential metabolites were identified between the YG group and the RG group, with 396 significantly upregulated and 81 significantly downregulated (Figure 6C–E).

**Figure 7:**
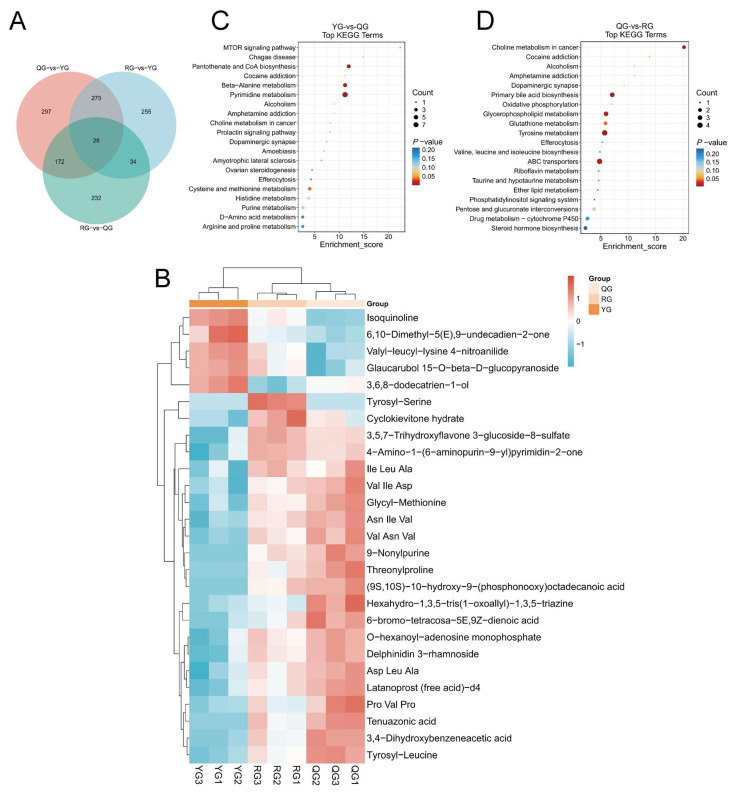
(**A**) Venn diagram of shared metabolites across treatment groups; (**B**) Clustered heatmap of 28 key metabolites; color gradient indicates relative abundance levels across groups. (**C**,**D**) KEGG pathway enrichment bubble charts (top 20 pathways): (**C**) YG vs. QG; (**D**) QG vs. RG; *Y*-axis: names of enriched metabolic pathways; *X*-axis: enrichment factor; bubble size: number of metabolites involved; color gradient: −log_10_ (*p*-value).

Additionally, 509 metabolites were identified across all three treatment groups, indicating that dietary energy levels have a significant impact on colonic metabolites (Figure 7A). Based on this, we further constructed a clustered heatmap of the 28 shared differential metabolites among the three comparison groups to visually illustrate the clustering patterns of metabolites under different treatment conditions (Figure 7B). The KEGG enrichment analysis revealed that the YG group and the QG group were significantly enriched in four pathways: pantothenate and CoA biosynthesis, beta-Alanine metabolism, cysteine and methionine metabolism, and the MTOR signaling pathway (Figure 7C). The QG group and the RG group were significantly enriched in six pathways: Choline metabolism in cancer, Tyrosine metabolism, Primary bile acid biosynthesis, ABC transporters, Glycerophospholipid metabolism, and Glutathione metabolism (Figure 7D).

**Figure 8:**
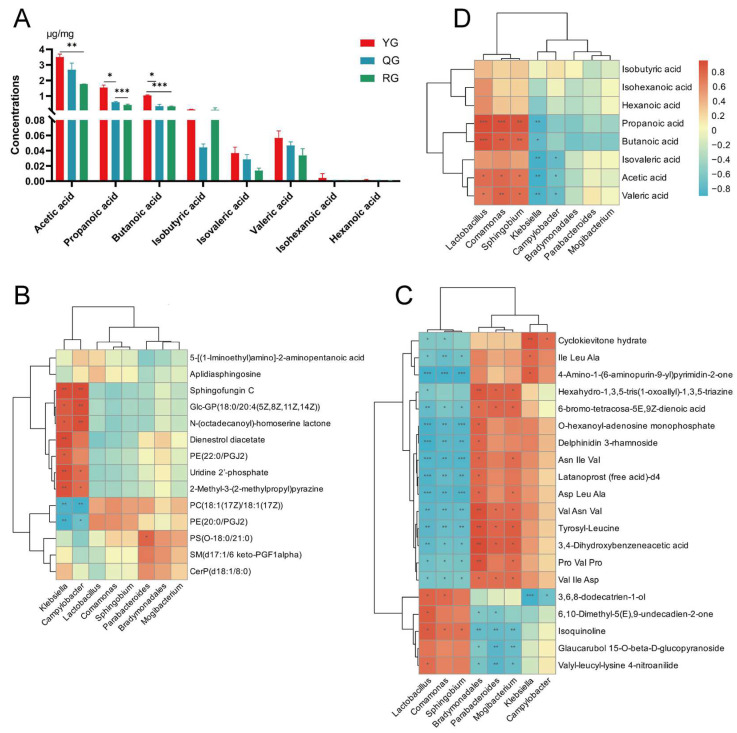
(**A**) Effects of various groups on the content of SCFAs in yak colonic digesta. From (left) to (right): Acetic acid, Propanoic acid, Isobutyric acid, Butanoic acid, Isovaleric acid, Valeric acid, Isohexanoic acid, and Hexanoic acid. Data are expressed as mean ± SD (*n* = 3). Significance levels: * *p* < 0.05, ** *p* < 0.01, *** *p* < 0.001. (**B**–**D**) Correlation heatmap between differential bacterial flora and serum differential metabolites, colonic differential metabolites, and SCFAs. (**B**) Rows and columns represent serum differential metabolites and differential bacterial flora. (**C**) Rows and columns represent colonic differential metabolites and differential bacterial flora. (**D**) Rows and columns represent SCFAs and differential bacterial flora; Cell color indicates the Spearman correlation coefficient. Significance levels: * *p* < 0.05, ** *p* < 0.01, *** *p* < 0.001.

In the targeted analysis of SCFAs in the colon, we detected significant differences in acetate, propionate, and butyrate across varying energy levels. The results showed that the YG group had significantly higher levels of acetate, propionate, and butyrate compared to the QG group and the high RG group (*p* < 0.05) (Figure 8A). This finding suggested that a low-energy diet may promote the accumulation of major SCFAs in the colon, potentially associated with alterations in microbial community structure and their substrate utilization patterns. Subsequently, we utilized the Spearman correlation coefficient to construct a correlation heatmap, analyzing the relationships between differential microbiota and serum and colonic differential metabolites as well as SCFAs. The results revealed that Klebsiella and Campylobacter exhibited significant positive correlations with multiple serum metabolites, such as Sphingofungin C, N-(octadecanoyl)-homoserine lactone, and Uridine 2′-phosphate (*p* < 0.05). We also found that they showed significant negative correlations with PC (18:1 (17Z)/18:1 (17Z)) and PE (20:0/PGJ2) (*p* < 0.05). Parabacteroides showed a significant positive correlation only with PS(O-18:0/21:0) (*p* < 0.05) (Figure 8B). As shown in Figure 8C, we found that *Lactobacillus*, *Comamonas*, and *Sphingobium* exhibited opposite trends compared to the other five differential microbial groups. *Bradymonadales*, *Mogibacterium*, *Parabacteroides*, *Campylobacter*, and *Klebsiella* showed significant positive correlations with most metabolites (*p* < 0.05). For instance, *Bradymonadales* exhibited a highly significant positive correlation with the majority of the differential metabolites (*p* < 0.01). *Klebsiella* exhibited a highly significant positive correlation with Cyclokievitone hydrate, lIle Leu Ala, and 4-Amino-1-(6-aminopurin-9-yl) pyrimidin-2-one (*p* < 0.05), and a highly significant negative correlation with 3,6,8-dodecatrien-1-ol (*p* < 0.001). *Parabacteroides* showed a significant positive correlation with Val Ile Asp and Tyrosyl-Leucine (*p* < 0.05), and a significant negative correlation with Isoquinoline and Valyl-leucyl-lysine 4-nitroanilide (*p* < 0.05). Subsequently, in the results of differential microbiota and SCFAs (Figure 8D), we observed the same trend as with the differential metabolites. For instance, significant positive correlations with acetic acid, propanoic acid, isovaleric acid, and valeric acid (*p* < 0.05) were observed in *Comamonas*, *Lactobacillus*, and *Sphingobium*. Conversely, *Klebsiella* demonstrated highly significant negative correlations with acetic acid, propanoic acid, isovaleric acid, and valeric acid (*p* < 0.05).

### 3.5. Response of Colon Transcriptomics to Diets with Different Energy Levels

**Figure 9:**
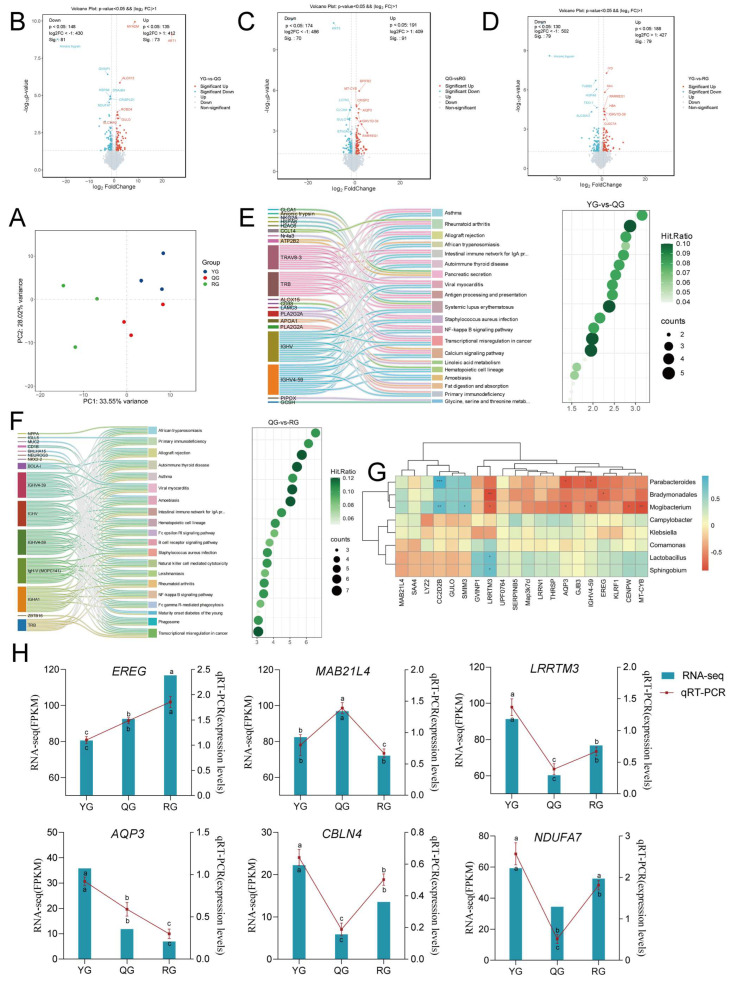
(**A**) PCA score plots of transcriptome data between the three comparison groups. (**B**) Volcano plots of differential genes: (**C**) YG vs. QG; (**D**) QG vs. RG; (**E**) YG vs. RG; Red dots represent significantly upregulated genes, blue dots represent significantly downregulated genes, and gray dots represent genes with no significant difference, with screening criteria of *p* < 0.05 and VIP > 1.0; (**E**,**F**) Sankey and bubble plots for pathway enrichment analysis. (**E**) YG vs. QG; (**F**) QG vs. RG; The Sankey plot (left) shows the relationship between differentially expressed genes and enriched KEGG pathways. Each ribbon represents the connection between a specific gene and its associated pathway. The bubble plot (**right**) summarizes the enrichment results, with the *x*-axis representing the enrichment score, bubble size indicating the number of genes involved in each pathway, and bubble color reflecting the pathway hit rate (proportion of genes). (**G**) Correlation heatmap between differential bacterial flora and differential genes. Rows and columns represent differential bacterial flora and differential genes, respectively, and cell color indicates the Spearman correlation coefficient. Significance levels: * *p* < 0.05, ** *p* < 0.01, *** *p* < 0.001. (**H**) Validation of RNA-Seq by qRT-PCR analysis. One-way ANOVA was used to analyze the differences between the groups, and Tukey’s HSD was used to assess statistical significance. Different lowercase letters (a, b, c) in the figures indicate significant differences (*p* < 0.05). Groups with the same lowercase letters or containing the same lowercase letters did not differ significantly. The letters above the line graph indicate the significance of RNA-seq, and the letters below the line graph indicate the significance of qRT-PCR.

The PAC of colonic transcriptomics showed separation among the three groups (Figure 9A). As illustrated in Figure 9B–D, 73 significantly upregulated genes and 81 significantly downregulated genes were screened between the YG group and the QG group, 91 significantly upregulated genes and 70 significantly downregulated genes were screened between the QG group and the RG group, and 79 significantly upregulated genes and 79 significantly downregulated genes were screened between the YG group and the RG group. Subsequently, we selected the top 20 significantly enriched pathways for visualization. The YG group and the QG group were significantly enriched in pathways such as Asthma, Rheumatoid arthritis, Allograft rejection, and African trypanosomiasis (*p* < 0.01) (Figure 9E). The QG group and the RG group exhibited highly significant enrichment in pathways such as African trypanosomiasis, Primary immunodeficiency, Allograft rejection, and Autoimmune thyroid disease (*p* < 0.001) (Figure 9F). Subsequently, we conducted a correlation analysis between the differentially expressed genes and the differential microbiota. As shown in Figure 9G, *Parabacteroides* and *CC2D2B* displayed a highly significant negative correlation (*p* < 0.001), while *Bradymonadales* and *LRRTM3* showed a significant positive correlation (*p* < 0.01). *Mogibacterium* and *CC2D2B* also showed a significant negative correlation (*p* < 0.01) and a significant positive correlation with *MT-CYB* (*p* < 0.01). To validate the transcriptomic data, we randomly selected six DEGs for the assessment of mRNA levels using qRT-PCR. As shown in Figure 9H, the qRT-PCR results showed that the expression patterns of the genes are consistent with the RNA-seq data, indicating that the transcriptomic results are reliable.

### 3.6. Multi-Omics Integration Analysis

**Figure 10:**
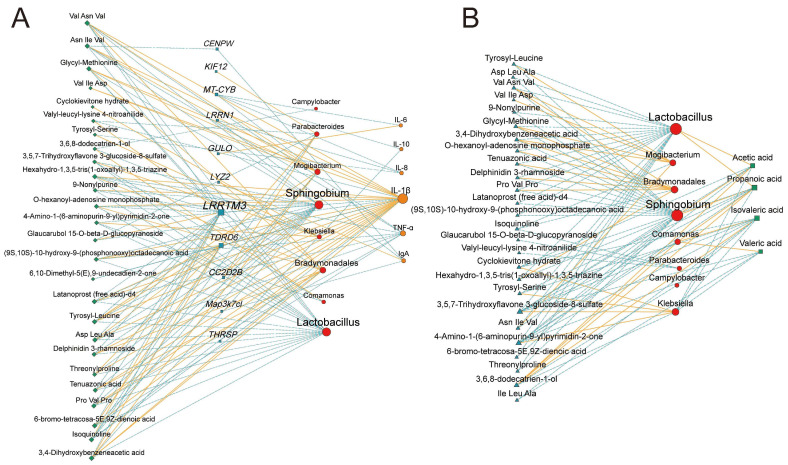
(**A**) The Spearman correlation network illustrates the relationships among differential microbiota, colonic differential metabolites, host genes, and colonic immune indicators. The color of edges: red, positive; blue, negative. Node colors: red circles, differential microbiota; blue squares, host genes; green diamonds, colonic differential metabolites; orange circles, colonic immune indicators. Only strong correlations were displayed (|r| >  0.5, *p*  <  0.05). (**B**) The Spearman correlation network illustrates the relationships among differential microbiota, colonic differential metabolites, and SCFAs. The color of edges: red, positive; blue, negative. Node colors: red circles, differential microbiota; blue triangles, differential colonic metabolites; green squares, SCFAs. Only strong correlations were displayed (|R| >  0.5, *p*  <  0.05).

Based on Spearman correlation analysis, we constructed association networks between differential microbiota and colonic differential metabolites, differential genes, and colonic immunity, as well as between differential microbiota and SCFAs (Figure 10). The results (Figure 10A) revealed multiple significant relationships involving *Sphingobium*, *Lactobacillus*, *Mogibacterium*, IL-1β, *LRRTM3*, and *TDRD6*. For instance, *Sphingobium* and *Lactobacillus* displayed negative correlations with several metabolites (−0.81 < R < −0.84, *p* < 0.05). *Comamonas* showed a significant negative correlation with 3,5,7-Trihydroxyflavone 3-glucoside-8-sulfate and 4-Amino-1-(6-aminopurin-9-yl) pyrimidin-2-one (R = −0.93, *p* < 0.05) (R = −0.90, *p* < 0.05), and a significant positive correlation with 3,6,8-dodecatrien-1-ol (R = 0.88, *p* < 0.05). LRRTM3 showed a negative correlation with multiple metabolites and IL-1β (−0.82 < R < −0.93, *p* < 0.05), and a significant positive correlation with Isoquinoline, Valyl-leucyl-lysine 4-nitroanilide, and Glaucarubol 15-O-beta-D-glucopyranoside (0.82 < R < 0.85, *p* < 0.05). *TDRD6* exhibited a positive correlation with multiple metabolites and IL-1β (0.82 < R < 0.88, *p* < 0.05). Subsequently, in Figure 10B, we observed that Acetic acid, Propanoic acid, Isovaleric acid, and Valeric acid exhibited significant relationships with multiple differential bacteria and differential metabolites (|R| > 0.50, *p* < 0.05). *Lactobacillus* and *Sphingobium* showed significant positive correlations with Acetic acid and Propanoic acid (0.82 < R < 0.84, *p* < 0.05). *Comamonas* demonstrated a significant positive correlation with Valeric acid (R = 0.90, *p* < 0.05).

## 4. Discussion

The colon plays a crucial role in energy metabolism and intestinal health of ruminants during various physiological stages [35,36,37,38]. As an important livestock species endemic to the plateau, the Pamir yak has long been exposed to extreme environments characterized by hypoxia, cold, and nutritional scarcity, making its reliance on energy metabolism particularly pronounced [39]. However, there is still a lack of in-depth understanding regarding the systemic response patterns of high-altitude yaks at the colon level under different energy supply conditions. Consequently, this study integrated multi-omics approaches and demonstrated that an appropriate dietary energy level may regulate colonic homeostasis through the microbe–metabolite–host interaction pathway, providing a new theoretical framework for understanding the energy-dependent intestinal adaptation mechanism of high-altitude yaks.

Our research indicated that high-energy feeding could enhance animal growth performance. But colonic pathological observations reveal disorganized glandular arrangement and increased inflammatory cell infiltration, suggesting that excessive energy supply may disrupt the colonic barrier structure and induce local inflammatory responses. These findings were consistent with studies in other ruminants [40,41]. In dairy cows, it has also been reported that high-concentration diets can induce an increase in the expression of inflammatory factors and cause inflammatory damage in cecal epithelial cells [42]. In this study, the levels of IgA, IgG, and IL-10 in the RG group were significantly decreased, further supporting that high-energy feeding may exacerbate intestinal inflammatory responses by affecting the host’s immune status. Furthermore, we observed a significant increase in IL-8 and IL-6 activity in QG animals. This phenomenon was similar to previous findings in weaned piglets [43]. Transient intestinal upregulation of inflammatory factors such as IL-8, accompanied by increased secretion of immune-related IgA, was considered an immune stress response to dietary and environmental changes. Therefore, we speculated that the elevation of inflammatory cytokines in the QG group may reflect an adaptive immune activation state in yaks during the transition from a low-energy to a high-energy diet. At this stage, the immune response is still under dynamic regulation, manifested as a transient enhancement of inflammatory signaling rather than the development of chronic inflammation or immunosuppression. However, in the RG group, the levels of IL-6 and IL-8 were markedly reduced. In the context of immunometabolism, this phenomenon may be associated with immune tolerance or negative feedback regulation induced by excessive energy supply. Previous studies have indicated that under moderate nutritional stimulation, immune cells undergo metabolic reprogramming (e.g., a shift from the TCA cycle to glycolysis) to enter an activated state, thereby leading to a moderate increase in pro-inflammatory cytokines. In contrast, when energy supply becomes excessive, the organism may initiate tolerance or suppressive mechanisms to prevent excessive immune activation and chronic inflammation [44,45,46]. This mechanism may explain the decreased levels of inflammatory cytokines observed in the RG group, suggesting that high energy intake exerts a suppressive regulatory effect on immune responses. These findings provided evidence at the colonic level in plateau yaks, highlighting the close connection between energy levels and intestinal immune homeostasis.

In the microbiome analysis, we observed that alterations in the microbiome at both taxonomic and functional levels were closely associated with gut health. Specifically, we observed a significant increase in *Klebsiella* and *Campylobacter* in the RG group, while the LeFSe analysis revealed no enrichment of typical probiotics. Previous studies have reported that *Klebsiella* serves as an opportunistic pathogen, frequently leading to severe infections in immunocompromised individuals [47,48,49,50]. In contrast, *Campylobacter* was recognized as a pathogen affecting both humans and animals and was among the primary causative agents of bacterial gastroenteritis [51,52]. Functional prediction results indicated that the RG group was exclusively enriched in the thyroid hormone synthesis pathway. This suggested that its microbial metabolic functions may be primarily associated with energy absorption, while playing a limited role in maintaining intestinal homeostasis [53,54]. Furthermore, correlation analysis revealed potential links between microbes and immune phenotypes. *Klebsiella* exhibited a negative correlation with TNF-α and IgG. This finding implied that the increase in potential pathogenic bacteria within the high-energy group could disrupt host immune function [55,56]. Meanwhile, elevated levels of IgA, IL-8, and IL-6 indicate that the host is actively responding to inflammation, which may help regulate pathogenic microbiota in the gut. In contrast, the QG group exhibited significant enrichment of three dominant bacterial taxa: *Bradymonadales* [57], *Parabacteroides* [58,59], and *Mogibacterium* [60]. Although these taxa demonstrated a positive correlation with IL-1β, functional enrichment analysis indicated that the QG group had significantly enhanced pathways related to carbon metabolism, ribosome function, nucleotide metabolism, and protein synthesis [61]. These findings were consistent with prior research highlighting the gut microbiome’s role as a pivotal regulator of host metabolism. They suggested that during periods of active growth, microbes facilitate physiological demands and maintain immune homeostasis by enhancing nucleotide, amino acid, and energy metabolism [62]. Notably, even low-abundance taxa such as *Lactobacillus* may play crucial roles in this process. Specifically, the enhancement of the nucleotide metabolic pathway suggested that the microbiota may synthesize and turnover nucleotides in the host, thereby fulfilling the demand for glycolysis and TCA cycle intermediates required for the proliferation and functional maintenance of immune cells [63]. Similarly, studies on sheep have demonstrated that dietary changes can activate glycolysis, the TCA cycle, and ATP generation pathways within the gut microbiota, thereby enhancing the host’s energy utilization efficiency [64]. These findings indicated that under medium-energy feeding conditions, the microbiota exhibits enhanced metabolic activity, which not only supported moderate animal growth but also helped maintain a dynamic balance between colonic microecology and host immunity to a certain extent.

Our study further investigated the role of microbiome alterations in influencing serum and colonic metabolites. The KEGG functional enrichment results indicated that, when comparing the YG group to the QG group, both the serum metabolome and microbial functional predictions exhibit significant enrichment in the pathways of pyruvate metabolism, as well as glycine, serine, and threonine metabolism. Firstly, pyruvate metabolism, as the central hub of glycolysis, the tricarboxylic acid cycle, and *lactobacillus* fermentation, directly determines the efficiency of energy generation and conversion [65]. Under low-energy conditions, the host may rely more on the efficient utilization of pyruvate to maintain basal metabolism, while the gut microbiota compensates for the host’s insufficient energy supply to some extent by regulating the metabolic direction of pyruvate towards Lactic acid, Acetic acid, or Propanoic acid [66]. Additionally, several of these metabolites exhibit enzymatic activity or signaling functions, suggesting their involvement in the repair, regeneration, and inflammation regulation of intestinal epithelial tissue. This provides potential support for the findings observed in our histopathological analysis. This mechanism was consistent with the previously reported conclusion that “gut microbiota provides an additional energy source for the host through the production of SCFAs” [67]. In line with this, we observed in targeted assays that the concentrations of Acetic acid, Propionic acid, and Butyric acid in the YG group were significantly higher than those in the QG and RG groups (*p* < 0.05). This further suggested that a low-energy diet may enhance the accumulation of SCFAs by promoting pyruvate and related metabolic pathways, thereby alleviating host energy deficiency and maintaining intestinal homeostasis. Secondly, the metabolism of glycine, serine, and threonine, which are essential branches of Amino acid metabolism, encompasses one-carbon metabolism, Nucleotide synthesis, and Protein synthesis. This process played a significant role in the proliferation of immune cells and the defense against oxidative stress, particularly through the synthesis of glutathione [68,69]. The significant enrichment of this pathway in the QG group suggested that moderate energy levels may be more conducive to supporting the maintenance of host anabolism and immune function. This balance provides substrates and energy support for the generation of SCFA, ultimately achieving a harmonious relationship between growth performance and intestinal health. Additionally, our findings revealed that both the colonic metabolome and microbial functional predictions were significantly enriched in the pantothenate and CoA biosynthesis pathway. This pathway serves as a fundamental component of coenzyme A synthesis, which was crucial not only for the generation of acetyl-CoA in the tricarboxylic acid cycle but also for enhancing the efficiency of fatty acid β-oxidation and the catabolism of specific amino acids. Studies have demonstrated that specific gut microbiota can enhance the host’s metabolic flexibility in energy-restricted environments by synthesizing derivatives of pantothenic acid, which in turn promotes the upregulation of CoA levels [70,71]. We hypothesized that, under conditions of low energy intake, the gut microbiota of yaks may maintain energy homeostasis and immune adaptability through the multi-pathway regulation of “pyruvate metabolism - amino acid metabolism - CoA synthesis.” This finding provides systematic evidence for elucidating the colonic metabolic regulation strategies of plateau ruminants under prolonged nutritional stress.

Furthermore, our findings elucidated the potential regulatory role of microbial communities in the mucosal immune status of the yak colon. The results indicated that the differentially expressed genes in the YG group and QG group were co-enriched in pathways associated with glycine, serine, and threonine metabolism, Fat digestion and absorption, as well as Antigen processing and presentation. This was demonstrated through both gene set enrichment analysis and microbial function prediction analysis. We found that the glycine, serine, and threonine metabolism pathway aligns with the results of the serum metabolome [72,73]. The enrichment of the fat digestion and absorption pathway indicated that the gut microbiota may regulate lipid metabolism or enhance the production of SCFAs, which provided direct energy for colonic epithelial cells. This observation was consistent with the enrichment trend of lipid-related differential metabolites in the serum metabolome [74]. More critically, the enrichment of the antigen processing and presentation pathway suggested that the differential microbiota may enhance immune signaling and antigen presentation functions by regulating host gene expression, thereby maintaining mucosal immune homeostasis [75]. Based on the results of our correlation analysis, we hypothesized that alterations in the microbiota not only influence the expression of key genes in the host but may also contribute to the stabilization of the host’s immune status by enhancing immune cell activation and the efficiency of antigen processing. This was in line with previous research regarding the role of gut microbiota in mediating immune homeostasis [76].

Finally, we constructed a multi-omics association network based on Spearman correlation analysis, which revealed the interactions between the colonic microbiota and various indicators. Our results indicated that *Sphingobium* and *Lactobacillus* were significantly negatively correlated with a range of metabolites, while *Comamonas* exhibited selective regulatory effects across different metabolites. Furthermore, the host key genes *LRRTM3* and *TDRD6* displayed both positive and negative correlations with metabolites and immune factors, suggesting that the colonic microbiota may play a role in immune signaling and the maintenance of metabolic homeostasis by regulating the expression of specific genes and the levels of metabolites. The analysis of SCFAs revealed significant correlations between Acetic acid, Propanoic acid, Isovaleric acid, and Valeric acid with various differential microbiota and metabolites. Notably, *Lactobacillus* and *Sphingobium* demonstrated positive correlations with both acetate and propionate, whereas *Comamonas* exhibited a significant positive correlation specifically with valerate. These results also indicated that the dominant microbiota could regulate the intestinal environment through the production of SCFAs. Overall, these multi-omics association results were highly consistent with the analyses of the serum metabolome, colon metabolome, and transcriptome. This consistency further emphasizes the central role of gut microbiota in coordinating host energy metabolism and immune responses across varying energy levels. This finding supported our hypothesis. However, it is important to acknowledge certain limitations. The findings were derived from association analyses, which do not establish causality. Future research could utilize metagenomic assembly, proteomics, or multi-omics integration studies to further elucidate the mechanisms through which energy levels influence hindgut homeostasis and immune function in yaks. And our study was the use of a single gene for qRT-PCR validation. Although this choice was based on precedent in related studies, future investigations should involve more genes to strengthen the validation of transcriptomic results.

## 5. Conclusions

In summary, this study systematically elucidated the regulatory role of dietary energy levels on the interaction network of colon microbiota, metabolites, and the host in plateau yaks. Our findings revealed that under medium-energy dietary conditions, immune factors such as IgA, IgG, IL-8, and IL-6 increase during this stage. The dominant microbiota, including *Bradymonadales*, *Parabacteroides*, and *Mogibacterium*, regulated the expression of key host genes and metabolite levels by promoting Amino acid, Nucleotide, and Lipid metabolism. This regulation effectively coordinates immune signaling and energy metabolism, forming an interaction pattern that is conducive to colon health. Overall, maintaining a medium-energy dietary level can preserve the balance between colon microecology and host metabolism, providing important scientific evidence for optimizing dietary energy supply and promoting intestinal health in plateau ruminants. These research results emphasize the critical role of dietary energy in shaping the composition of intestinal flora and regulating the host’s immune and metabolic responses. Furthermore, they suggest that in plateau environments, the reasonable regulation of dietary energy intake is essential for ensuring animal health and growth.

## Data Availability

The datasets presented in this study could be found in online repositories. All raw sequence data were deposited in the NCBI Sequence Read Archive database under accession number PRJNA1313624.

## Supplementary Materials

The following supporting information can be downloaded at https://www.mdpi.com/article/10.3390/ani15192929/s1. Table S1: The feed ingredients and nutrient composition for yak diets. File S1: Untargeted metabolomics and Short-chain fatty acid determination.
