# Supplementary material for: Microbiota–Metabolite–Host Crosstalk Mediates the Impact of Dietary Energy Levels on Colonic Homeostasis in High-Altitude Ruminants
Source: Animals (Basel). 2025 Oct 9;15(19):2929. doi: 10.3390/ani15192929 (PMC12524189; doi:10.3390/ani15192929)
Supplement: Supplementary file 1 [file animals-15-02929-s001.zip › animals-3880958-supplementary/Supplementary Files/Supplementary File S1.pdf]

1 Untargeted metabolomics

Add 100 mg of the sample to a 1.5 mL centrifuge tube, along with a 6 mm grinding bead and 400 μL of a methanol-water mixture (4:1, v/v) containing L-2-chlorophenylalanine at a concentration of 4 μg/mL. Cool the mixture in a -40 °C freezer for 2 minutes, then transfer the centrifuge tube to a grinder and process at 60 Hz for 2 minutes. After grinding, perform ultrasonic extraction in an ice-water bath for 10 minutes, followed by incubation at -40 °C for 30 minutes. Centrifuge the mixture at 1200 rpm for 10 minutes at 4 °C. Transfer 300 μL of the supernatant to an LC-MS sample vial and allow it to evaporate completely. Reconstitute the dried residue with 300 μL of a methanol-water solution (1:4, v/v). Vortex for 30 seconds and sonicate in an ice-water bath for 3 minutes. Allow the solution to stand at -40 °C for 2 hours, then centrifuge at 1200 rpm for 10 minutes at 4 °C. Collect 150 μL of the supernatant and filter it through a 0.22 μm organic phase syringe filter. Transfer the filtrate to a vial for liquid chromatography injection and store at -80 °C for subsequent liquid chromatography-mass spectrometry analysis. Additionally, prepare quality control samples by mixing equal volumes of all samples to monitor the stability of the analysis.

The analytical instrument employed in this experiment was an LC/MS system, which comprised an ACQUITY UPLC I-Class+ ultra-performance liquid chromatograph coupled with a QE+ high-resolution mass spectrometer featuring a heated electrospray ionization (ESI) source (Thermo Fisher Scientific, Waltham, MA, USA). This setup was utilized for analyzing metabolic profiles in both ESI positive and ESI negative ion modes. An ACQUITY UPLC HSS T3 column (1.8 μm, 2.1 × 100 mm) was employed in both ion modes. The gradient elution system included (A) water (containing 0.1% formic acid, v/v) and (B) acetonitrile (containing 0.1% formic acid, v/v), with the gradient defined as follows: 0.01 min, 5% B; 2 min, 5% B; 4 min, 30% B; 8 min, 50% B; 10 min, 80% B; 14 min, 100% B; 15 min, 100% B; 15.1 min, 5% B; and 16 min, 5% B. The flow rate was set at 0.35 mL/min, and the column temperature was maintained at 45 °C. All samples were kept at 10 °C during the analysis, with an injection volume of 2 μL. The mass range analyzed was from 100

m/z to 1200 m/z. The first-stage mass spectrometry scan resolution was 70,000, while the second-stage mass spectrometry scan resolution was 17,500, with collision energies of 10, 20, and 40 eV. The mass spectrometer operated under the following conditions: spray voltage at 3800 V (+) and 3200 V (-); sheath gas flow rate at 35 arbitrary units; auxiliary gas flow rate at 8 arbitrary units; and capillary temperature at 320 °C.

The LC-MS data underwent baseline filtering, peak detection, integration, retention time correction, peak alignment, and normalization using Progenesis QI V2.3 (Nonlinear Dynamics, Newcastle, UK). Key parameters included a precursor ion tolerance of 5 ppm, a product ion tolerance of 10 ppm, and a product ion threshold of 5%. Compound identification was based on the accurate mass-to-charge ratio (m/z), secondary fragmentation spectra, and isotopic distribution, with annotations referencing the Human Metabolome Database (HMDB), LipidMaps (V2.3), Metlin, and a self-constructed database. The extracted data were further processed by excluding peaks with missing values exceeding 50% (ion intensity = 0) within the group. Zero values were replaced with half of the minimum detected value, and compounds were filtered based on qualitative identification results. Metabolites with database matching scores below 36 (out of a total score of 80) were considered unreliable and excluded. Finally, data from both positive and negative ion modes were merged into a single data matrix.

2 Short-chain fatty acid determination

Standard solution preparation

First, prepared the mixed standard by placing 9840 μL of n-butanol (HPLC grade) into a 15 mL centrifuge tube. Sequentially added the appropriate amounts of each of the eight short-chain fatty acid standards, and vortex the solution to ensure thorough mixing. This process will yield a mixed standard stock solution A for the eight short-chain fatty acids. To prepare the internal standard, add 9990 μL of HPLC-grade n-butanol to a 15 mL centrifuge tube. Then, incorporate 10 μL of the internal standard, 2-ethylbutyric acid, and vortex the mixture thoroughly to ensure complete mixing. This procedure will yield the internal standard stock solution B. The stock solution concentrations are as follows: Acetic acid (6000 μg/mL), Propanoic acid (3000 μg/mL), Butanoic acid (3000 μg/mL), Isobutyric acid (1500 μg/mL), Valeric acid (500 μg/mL), Isovaleric acid (1000 μg/mL), Hexanoic acid (200 μg/mL), Isohexanoic acid (500 μg/mL), 2-ethylbutyric acid (1000 μg/mL). The mixed standard A and B solutions were diluted with n-butanol to form 7 working solutions with different concentrations, and then placed into injection vials for GC-MS analysis.
