# Supplementary material for: Microbiota–Metabolite–Host Crosstalk Mediates the Impact of Dietary Energy Levels on Colonic Homeostasis in High-Altitude Ruminants
Source: Animals (Basel). 2025 Oct 9;15(19):2929. doi: 10.3390/ani15192929 (PMC12524189; doi:10.3390/ani15192929)
Supplement: Supplementary file 1 [file animals-15-02929-s001.zip › animals-3880958-supplementary/Supplementary Files/Table S1.pdf]

Table S1 The feed ingredients and nutrient composition for the yak diets of YG (1.53 MJ/kg NEG), QG (2.12 MJ/kg NEG), and RG (2.69 MJ/kg NEG).

Item	**Content (%)**		
	YG	QG	RG
Ingredient composition			
Maize	21	29.8	34.4
Cottonseed meal	3.25	4.6	5
Soybean meal	4.24	6.0	6.5
Soybean oil	0	0	0.6
Wheat bran	0	5.8	1
Molasses	0.92	1.3	0
Premix②	1.77	2.5	2.5
Wheat straw	0	50	50
Alfalfa hay	64.67	0	0
Nutritional composition①			
CP（%）	6.30	9.85	10.92
NEg (MJ/Kg)	1.53	2.12	2.69
EE（%）	2.83	2.46	3.09
ADF（%）	12.0	5.18	4.51
NDF（%）	50.2	43.42	41.84
Ca	0.75	1.62	1.59
P	0.26	0.55	0.54

Note：

① The values for NEg are the calculated values (NRC,2007), others were measured value.

② The premix provided the following per kg of diets: Fe 2500mg, Zn 1000mg, Cu 1000mg, Mn 1000mg, Se 7.5mg, I 20mg, VA 300000IU, VD 5000IU, VE 4000IU.

③ CP is Crude Protein, NEg is Net Energy, ADF is Acid Detergent Fiber, NDF is Neutral Detergent Fiber, Ca is Calcium, P is Phosphorus.
